# Assessing the performance of large language models in literature screening for pharmacovigilance: a comparative study

**DOI:** 10.3389/fdsfr.2024.1379260

**Published:** 2024-06-27

**Authors:** Dan Li, Leihong Wu, Mingfeng Zhang, Svitlana Shpyleva, Ying-Chi Lin, Ho-Yin Huang, Ting Li, Joshua Xu

**Affiliations:** ^1^ Division of Bioinformatics and Biostatistics, National Center for Toxicological Research, U.S. Food and Drug Administration, Jefferson, AR, United States; ^2^ Division of Epidemiology, Office of Surveillance and Epidemiology, Center for Drug Evaluation and Research, U.S. Food and Drug Administration, Silver Spring, MD, United States; ^3^ Division of Biochemical Toxicology, National Center for Toxicological Research, U.S. Food and Drug Administration, Jefferson, AR, United States; ^4^ School of Pharmacy, College of Pharmacy, Kaohsiung Medical University, Kaohsiung, Taiwan; ^5^ Master/Doctoral Degree Program in Toxicology, College of Pharmacy, Kaohsiung Medical University, Kaohsiung, Taiwan; ^6^ Department of Pharmacy, Kaohsiung Medical University Hospital, Kaohsiung Medical University, Kaohsiung, Taiwan

**Keywords:** pharmacovigilance, large language models, LLMs, literature based discovery, artificial intelligence

## Abstract

Pharmacovigilance plays a crucial role in ensuring the safety of pharmaceutical products. It involves the systematic monitoring of adverse events and the detection of potential safety concerns related to drugs. Manual literature screening for pharmacovigilance related articles is a labor-intensive and time-consuming task, requiring streamlined solutions to cope with the continuous growth of literature. The primary objective of this study is to assess the performance of Large Language Models (LLMs) in automating literature screening for pharmacovigilance, aiming to enhance the process by identifying relevant articles more effectively. This study represents a novel application of LLMs including OpenAI’s GPT-3.5, GPT-4, and Anthropic’s Claude2, in the field of pharmacovigilance, evaluating their ability to categorize medical publications as relevant or irrelevant for safety signal reviews. Our analysis encompassed N-shot learning, chain-of-thought reasoning, and evaluating metrics, with a focus on factors impacting accuracy. The findings highlight the promising potential of LLMs in literature screening, achieving a reproducibility of 93%, sensitivity of 97%, and specificity of 67% showcasing notable strengths in terms of reproducibility and sensitivity, although with moderate specificity. Notably, performance improved when models were provided examples consisting of abstracts, labels, and corresponding reasoning explanations. Moreover, our exploration identified several potential contributing factors influencing prediction outcomes. These factors encompassed the choice of key words and prompts, the balance of the examples, and variations in reasoning explanations. By configuring advanced LLMs for efficient screening of extensive literature databases, this study underscores the transformative potential of these models in drug safety monitoring. Furthermore, these insights gained from this study can inform the development of automated systems for pharmacovigilance, contributing to the ongoing efforts to ensure the safety and efficacy of pharmacovigilance products.

## 1 Introduction

Pharmacovigilance, the science of monitoring and ensuring the safety of pharmaceutical products post market, stands as a vital cornerstone in the realm of public health and medicine (Beninger, 2018; Maqbool et al., 2019). It encompasses the diligent surveillance of adverse events, the detection of potential safety signals, and the evaluation of data from a wide array of sources, including scientific literature, regulatory reports, and patient feedback (Böhm et al., 2016; Hoffman et al., 2016). Yet, as the volume of medical literature continues to burgeon, manual screening of this extensive corpus remains an onerous and time-consuming task. The need for efficient and accurate tools to assist pharmacovigilance efforts remains substantial. Traditional computational methods like pattern matching, topic modeling, text mining and classification, etc., have been employed to review and filter the literature (Cohen et al., 2006; Burgard and Bittermann, 2023). However, these methods face several challenges, such as their inability to generalize across multiple tasks and adapt to new, unseen conditions, which prevents them from fully meeting the demands of pharmacovigilance.

In addition to the conventional computational methods, recently a set of research have used small-scale deep learning model, especially Bidirectional Encoder Representations from Transformers (BERTs), to facilitate literature screening. For example, Hassain et al., used BERT to detect adverse drug reactions in PubMed abstracts (Hussain et al., 2021); Wu et al., developed NLP-DILI model, a predictive model based on BERT to find drug-induced liver injury relevant documents from FDA drug labeling (Wu et al., 2021) In a previous study, we have also developed BERT models to predict relevant literature for chlorine efficacy, safety (Wu et al., 2023), and Neurotoxicity relevance with COVID-19 (Wu et al., 2022). In general, models based on BERT outperform conventional computational methods (Aum and Choe, 2021; Sun et al., 2021), however, the key limitation has not been resolved; the models are still highly task-specific and can rarely be applied to a new task of literature screening. For example, a model developed for DILI relevance prediction will not perform well to find cardiotoxicity relevant documents, unless further fine-tuned or training with a large size of new collected data relevant to the new task. Recent strides in the field of Artificial Intelligence (AI), particularly in the domain of Large Language Models (LLMs), offer a promising solution to this challenge (Zhao, 2023). Models such as GPT-3.5 (OpenAI, 2023a), GPT-4 (OpenAI, 2023b), and Anthropic’s Claude2 (Anthropic, 2023) have demonstrated remarkable capabilities in natural language processing (NLP) and understanding. They have excelled in diverse NLP tasks, including text classification, summarization, translation, and more (Sun, 2023; Zhao, 2023).

Despite the advances in AI and NLP, the application of these technologies in pharmacovigilance remains fraught with challenges. The sheer volume and complexity of medical literature necessitate models that can not only process large datasets but also accurately identify relevant information amidst a plethora of unrelated data. Moreover, the dynamic nature of safety signals and the need for timely detection further compound the difficulty of automating literature screening. Ensuring the reproducibility and reliability of these models is also crucial, as any inaccuracies can have significant implications for public health and safety.

Given these challenges, this study aims to explore the transformative potential of LLMs in automating the screening of medical literature for pharmacovigilance purposes. Our primary objective is to assess the performance of these models in accurately categorizing medical publications and identifying drug safety signals amidst the extensive and ever-growing body of medical literature. To this end, we employ a task-specific, pre-labeled chlorine safety abstract dataset to assess the performance of LLMs for relevant abstracts prediction and filtering. Our investigation encompasses various facets of performance, such as prompt, N-shot learning, chain-of-thought reasoning, sensitivity, specificity, and factors influencing accuracy.

With a thoughtfully designed prompt and some examples comprising abstracts, labels, and corresponding reasoning explanations, we achieved high sensitivity (97%) alongside moderate specificity (67%) within a group of 40 positive and 160 negative samples. A comparison with scenarios where no reasoning was provided revealed a slight performance increase with a higher number of reasoning explanations. Notably, the models exhibited consistent performance across diverse sets of reasoning explanations, resulting in a high level of reproducibility (93%). These results affirm the reliability of LLMs for literature screening, suggesting their trustworthiness in the context of our study and their potential for assisting humans in related tasks. Furthermore, this framework has the potential to be extended to other pharmacovigilance tasks, given that appropriate examples or contexts are provided to the models.

In this study, our exploration of LLMs for literature screening in pharmacovigilance underscores their significant potential. The observed performance highlights their efficacy in automating the screening process. While we emphasize the immediate application of LLMs for literature analysis, it's essential to recognize the broader implications. The synergy of LLMs and advanced AI in pharmacovigilance could lead to transformative advancements, ultimately enhancing patient safety and reshaping healthcare practices. However, these promising prospects necessitate further validation in real-world applications to fully unleash the revolutionary impact of AI in pharmacovigilance.

## 2 Methods

### 2.1 The preparation of example set and testing sets

In our previous study (Wu et al., 2023), we curated a dataset consisting of research abstracts, systematically categorizing them as either relevant or irrelevant to the topic of chlorine safety. These labels were established through expert evaluation and consensus. From this dataset, we randomly selected a subset of 30 abstracts, 15 relevant and 15 irrelevant, with 200–300 words as the example set. This set served as valuable background information for LLMs to refer. Based on these example abstracts and their labels, we prepared multiple sets of reasoning explanations, employing both human experts and LLMs including GPT-3.5, GPT-4, Claude2, to facilitate comparative analyses. We presented the models with the abstracts one by one and asked: “This abstract is considered as relevant/irrelevant to my chlorine safety study—you must take this as true label. Please provide me the reasoning process in around 100 words”. The models also need to consider the provided labels as true. This approach allowed us to evaluate and contrast the interpretability of the reasoning provided by the models against human-generated explanations.

Two testing sets were prepared by randomly collecting 40 (20 relevant and 20 irrelevant) and 200 (40 relevant and 160 irrelevant) abstracts, ensuring no overlap with each other or the example set. We excluded abstracts that were either too short or too long from the selection, ensuring that the final abstracts were within the range of 200–300 words. We asked the models to review the testing abstracts and predict their categories with certain numbers of relevant and irrelevant examples provided to the model to facilitate a N-shot learning scenario (Wang et al., 2020). Each example consisted of the original abstract, the pre-defined label, and reasoning. The prediction process for the testing set was repeated 10 times to measure reproducibility and other metrics comprehensively. The primary results in this study were based on an optimized prompt for our dataset; more details will be discussed later in this paper.

### 2.2 The calculation of all the metrics

Reproducibility was calculated between two runs (different combinations of examples provided to models) as the number of identical predictions divided by the total abstracts were predicted, either 40 or 200 in our study. Then, all the paired reproducibility were put together and plotted.

$$Reproducibility=\frac{\sum_{i=1}^{N}\left({Prediction\;RunA}_{i}={Prediction\;RunB}_{i}\right)}{N}$$

Where *N* is the total number of abstracts for prediction. *Prediction RunA*_*i*_ and *Prediction RunB*_*i*_ are the prediction results of the *i*th abstract in RunA and RunB, respectively.

Sensitivity and specificity were calculated as:

$$Sensitivity=\frac{Number\;of\;correctly\;predicted\;relevant\;abstracts}{Total\;prelabeled\;relevant\;abstracts}$$

$$Specificity=\frac{Number\;of\;correctly\;predicted\;irrelevant\;abstracts}{Total\;prelabeled\;irrelevant\;abstracts}$$

In this study, we had 20 and 40 pre-labelled relevant abstracts, and 20 and 160 irrelevant abstracts in the two testing sets.

Precision was calculated as:

$$Precision=\frac{Number\;of\;correctly\;predicted\;relevant\;abstracts}{Total\;number\;of\;abstracts\;predicted\;as\;relevant}$$

And F1-score as:

$$F1score=2*\;\frac{Precision*Sensitivity}{Precision+Sensitivity}$$

## 3 Results

**FIGURE 1:**
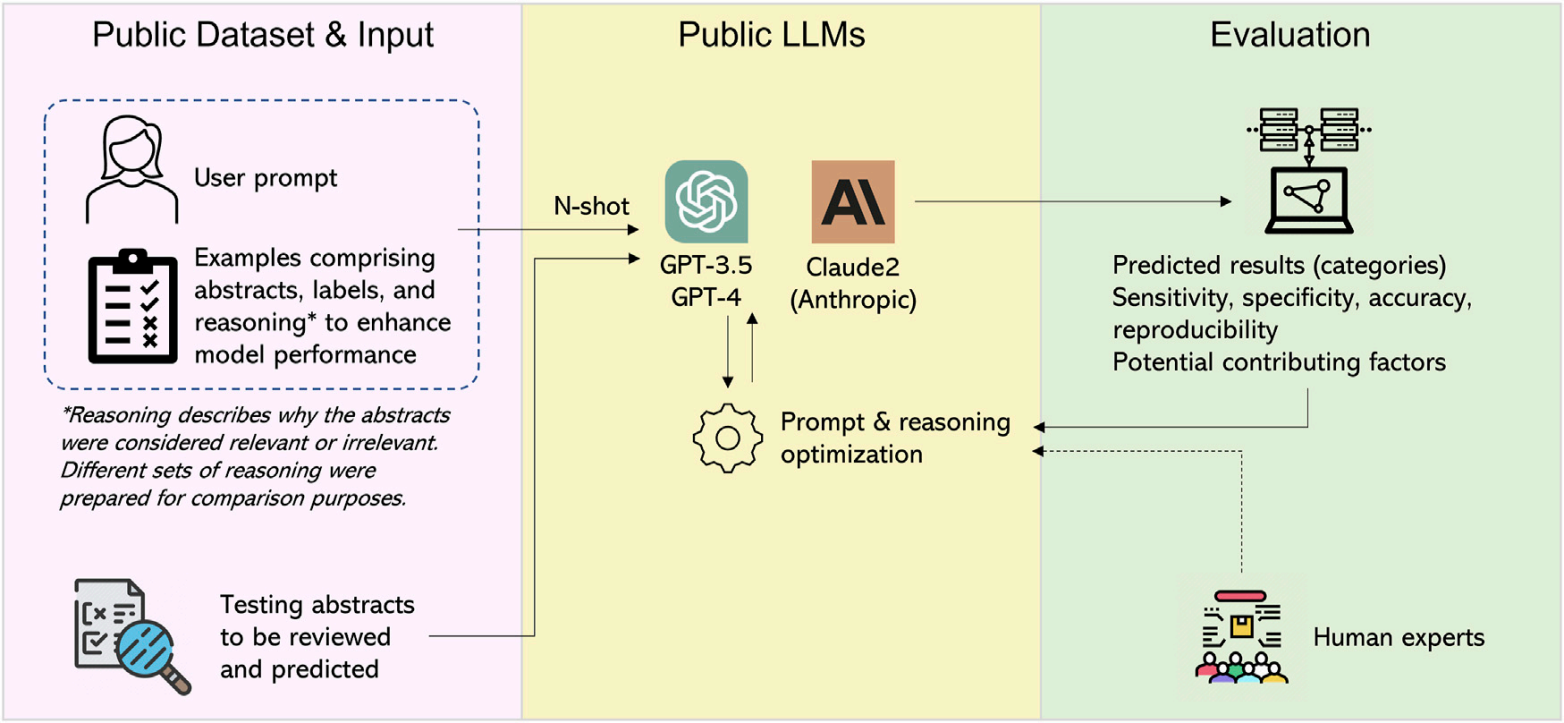
Study design.

In this study, our primary objective was to evaluate the performance of LLMs and enhance their effectiveness by optimizing the prompts and providing reasoning examples, with a specific focus on GPT-3.5 model equipped with an efficient Application Programming Interface (API) efficiently available. Our aim was to classify research abstracts as either relevant or irrelevant to chlorine safety study (Wu et al., 2023). To this end, we conducted a detailed study with several key elements illustrated in Figure 1. We crafted and refined multiple user prompts to communicate effectively with the LLMs to obtain optimal outcomes. We employed various metrics, including reproducibility, sensitivity, specificity, precision, and F1-score. These were calculated based on the predicted results and thoroughly evaluated with two distinct sets of testing abstracts (Methods). Throughout the optimization processes, experienced human experts actively engaged in evaluating and engineering the prompt, reasoning, and other settings to achieve enhanced performance.

### 3.1 Overall performance assessment

**FIGURE 2:**
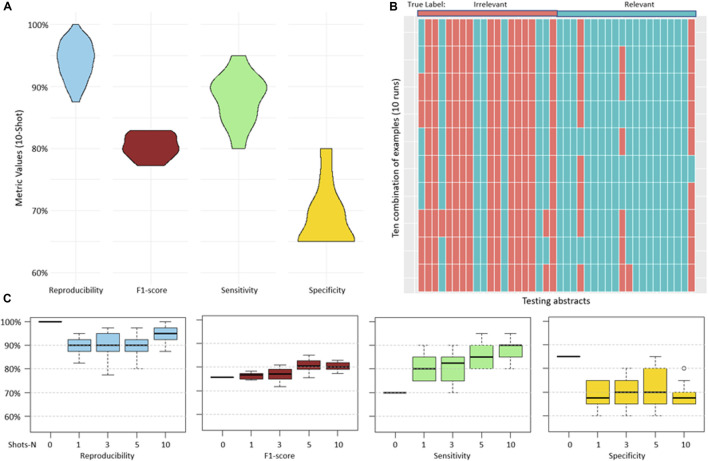
**(A)**, overview of the abstract prediction result with 10-shot learning and human expert generated reasoning provided to the model. **(B)**, prediction results of individual abstracts in each run (10 runs in total). **(C)**, performance differences across varying numbers of shots on reproducibility, sensitivity, specificity, and precision. There was no reasoning explanation provided in 0-shot learning, the input was identical for 10 runs, resulting in an identical prediction outcome.

For each testing abstract, a human expert provided reasoning explanations that detailed why the abstracts were categorized as relevant or irrelevant. With this, high reproducibility with a median of 95% and sensitivity with a median of 90% were achieved. However, specificity and precision were lower, achieving median values of 67.5% and 73.5% respectively (Figure 2A). The high reproducibility indicated that the model consistently produced similar responses across different examples provided. Figure 2B showed a detailed breakdown of predicted categories for each testing abstract in each run. Notably, certain abstracts consistently received incorrect predictions across all 10 rounds. We speculated that this could be due to variations in the model’s interpretation or potential complexities inherent in the original abstracts (Discussion).

Next, our investigation extended to comparing the performance of GPT-3.5 across various few-shot scenarios: 0-shot, 1-shot, 3-shot, 5-shot, and 10-shot (Wang et al., 2020). In the 0-shot scenario, the model was given no prior examples. For the other scenarios, a varying number of positive and negative examples were provided, each accompanied by abstracts, labels, and corresponding reasoning. In this study, we collected a dataset of 30 abstracts as examples. Specifically, in scenarios like 10-shot learning, the same example abstracts were sometimes used across different runs.

We conducted a comprehensive evaluation of reproducibility, sensitivity, specificity, and precision across these different shot settings. Generally, we observed a trend where increasing the number of shots led to a gradual improvement in all metrics, though the improvements were not significant. The metrics showed a narrower range with more shots, reflecting a stable performance by the model. The overall observation suggests that the results became more stable with an increase in the number of shots, emphasizing the potential benefits of providing additional examples to models (Figure 2C).

### 3.2 Reasoning explanations exert a subtle influence on performance

**FIGURE 3:**
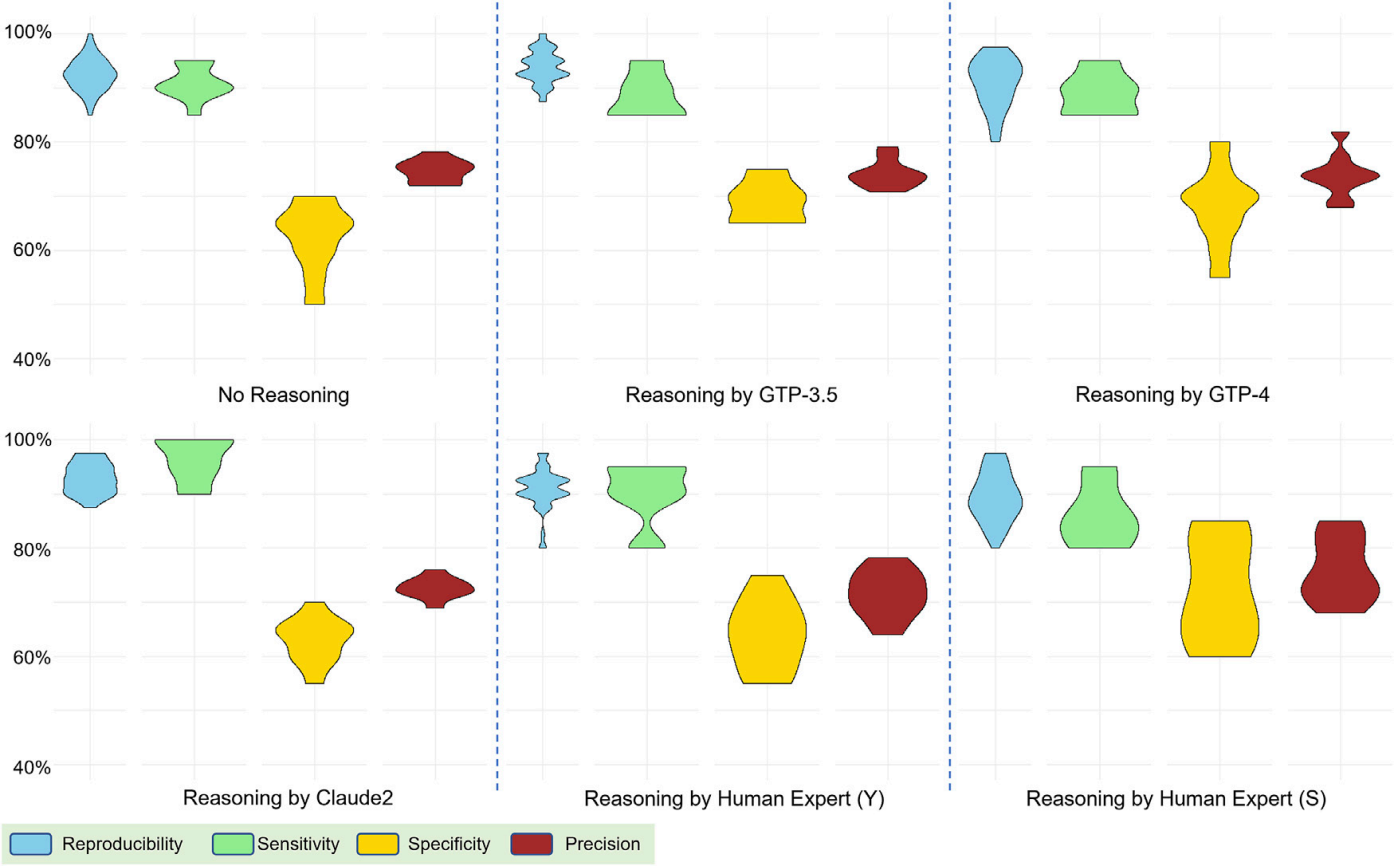
Performance comparison based on reasoning explanations from various sources: generated by human experts and AI models. The evaluation involved four metrics: reproducibility, sensitivity, specificity, and precision.

In our exploration of the impact of reasoning explanations within a 5-shot learning scenario, where five examples were provided to the model, we compared the performance differences across reasoning explanations generated by four human experts and various models, including Claude2, GPT-3.5, and GPT-4. These human experts have different backgrounds and reviewed the example abstracts independently. They were required to accept the predefined labels and generate reasoning explanations based on them. The ten runs of predictions were based on fixed combinations of examples so that the results were comparable for different reasoning sets. Surprisingly, we found that incorporating reasoning explanations resulted in only a marginal increase in the measured metrics. We observed no significant improvement when compared to scenarios with no reasoning provided (only abstracts and labels). (Figure 3). Additionally, we conducted comparative analyses between human-generated and model-generated reasoning explanations, contributing to a nuanced understanding of the model’s interpretability and alignment with domain-specific knowledge. Unexpectedly, the introduction of human-generated reasoning did not yield a discernible enhancement; instead, we observed a broader range in metrics, indicating a potential decrease in performance compared to AI-generated reasoning. These intriguing findings underscore the nuanced dynamics in the impact of reasoning explanations on model performance in the context of our 5-shot learning experiments (Discussion).

### 3.3 Reasoning quality evaluation: GPT-4’s perspective

In a meticulous examination of reasoning quality, we engaged GPT-4 in a discerning evaluation task. Presenting each of the 30 examples individually, we provided matched labels and reasoning explanations by one of the human experts, GPT-3.5, GPT-4, and Claude2 to GPT-4. The objective was to assess which reasoning aligned best with a given abstract from GPT-4’s perspective. The results revealed a noteworthy observation, as GPT-4 consistently identified reasoning explanations by Claude2 as the most fitting for 24 out of the 30 abstracts. Remarkably, for the other six abstracts, the human, GPT-3.5, and GPT-4 performed equally, with each generating two of the best reasoning explanations. In addition, we asked GPT-4 to generate the judgement statements for the best reasoning of each example abstract. These statements offer valuable insights into the optimal design of prompts and reasoning strategies for LLM for improving performance. Moreover, high-quality reasoning explanations provided by LLMs hold the potential to enhance the decision-making efficiency and accuracy of human reviewers in their review tasks (Discussion).

### 3.4 Performance difference per distinct prompts

**FIGURE 4:**
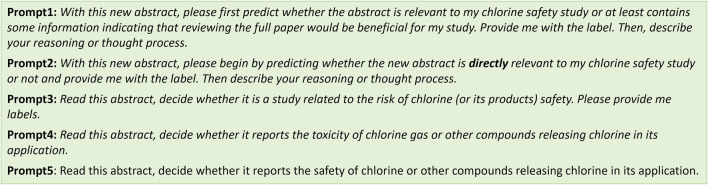
Five showcase prompts used in this study, among many that we tested. Background information and examples (abstract, label, reasoning) were provided to the models before presenting these prompts.

Recognizing the critical role prompts play in influencing model behavior (Wang, 2023; White, 2023), the prompt used in our primary analysis underwent careful design and optimization, achieving our goal with high sensitivity and reasonable specificity (Discussion). In this section, we showcase five of the prompts tested (Figure 4) to highlight their diverse impacts on LLM performance. The detailed results of these supplementary prompt analyses are presented in Table 1, offering insights into the dynamic interplay between prompts and LLM performance.

**TABLE 1:** The prediction performances of GPT-3.5 and GPT-4 based on distinct prompts.

Prompt	Predicting Model	Sensitivity (%)	Specificity (%)	Precision (%)	F1-score (%)	Accuracy (%)
#1	GPT-3.5	95	45	63.3	76	70
#2	GPT-3.5	95	50	66.1	78.8	72.5
#3	GPT-3.5	97.5	65	72.5	83	80
#4	GPT-3.5	87.5	65	72	80	77.5
#5	GPT-3.5	97.5	55	67.9	79.2	75
#1	GPT-4	95	62.5	71.7	82.6	80
#2	GPT-4	90	65	73.1	81.7	78.8
#3	GPT-4	90	65	72	80	77.5
#4	GPT-4	60	85	80.6	69.4	72.5
#5	GPT-4	80	70	72.7	76.7	75

We initially provided a brief introduction to the background information, informing the model about our research focus on chlorine safety. This was followed by the presentation of examples. Subsequently, we employed distinct prompts to conduct the prediction process with the models. See the Supplementary Material for the full prompt used in this study. We calculated and compared metrics associated with the five distinct prompts. Prompt1 underwent the initial testing for performance assessment. In prompt2, we tasked the model with directly identifying abstracts relevant to our chlorine safety study. For prompt3 and prompt4, our aim was to utilize the contextual understanding power of LLMs. We formulated generic questions that inquired whether the abstracts were related to chlorine safety or not. We conducted prompt5 by slightly modifying prompt4, changing the keyword “toxicity” to “safety” and removing “gas” from the sentence, to monitor the potential impacts of these different expressions. With these prompts, both GPT-3.5 and GPT-4 APIs were used to perform the predictions under a 5-shot learning scenario, using Claude2 reasoning (Table 1). Remarkably, prompt3 achieved the best performance when GPT-3.5 was used for prediction in this study and used for the primary analysis (Table 1).

Using the same prompts and reasoning for predictions, GPT-4 showed different patterns of metrics (Table 1; Supplementary Figure S1). For example, prompt3 did not reveal the best performance for GPT-4, particularly in sensitivity, which is a crucial metric for our study. Interestingly, when prompted with prompt4, we observed a significant drop in sensitivity but as high as 85% in specificity. Nevertheless, the results highlighted the potential for distinct performances among different models, even with the same prompt. It underscores the importance of being attentive to variations in outcomes when working with different LLMs.

### 3.5 Extended analysis to additional dataset

**FIGURE 5:**
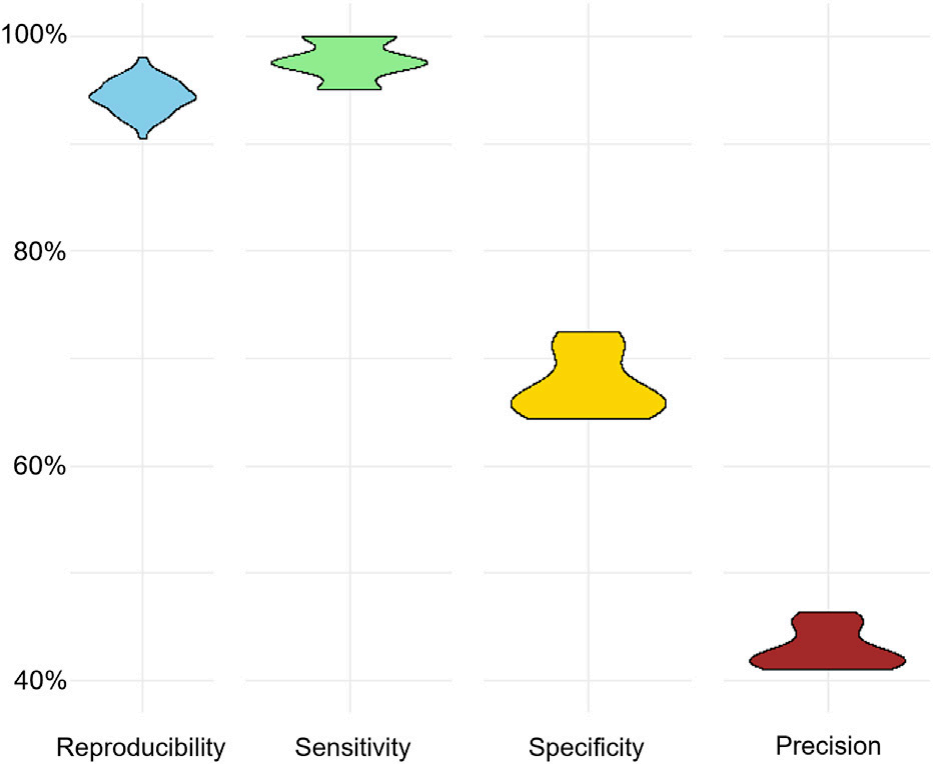
Evaluation of performance on an extended testing dataset comprising 200 abstracts (40 relevant and 160 irrelevant).

Building upon the optimal reasoning set generated by Claude2, we extended our inquiry to an additional testing set of 200 abstracts (40 relevant and 160 irrelevant abstracts) from the same collection of chlorine safety study abstract, without any overlap with the previous set of 40 abstracts. Still utilizing GPT-3.5 model and 5-shot learning scenario for categorization, the results demonstrated remarkable consistency with our initial observations, but with a lower precision (Figure 5). The precision, which gauges the accuracy of positive predictions among all predicted positives, is sensitive to imbalances in the dataset. In our extended dataset with more negatives, the number of false positives could increase, impacting precision and consequently leading to a lower precision. In summary, these findings bolster confidence in the reliability of LLMs, such as GPT-3.5, for efficiently conducting literature reviews. Accurate identification of relevant abstracts and effective filtering of a significant portion of irrelevant content demonstrate the efficacy of these models, ultimately reducing the burden on human reviewers.

## 4 Conclusion

This study delves into the realm of AI-driven pharmacovigilance, specifically employing LLMs for automated literature screening. Evaluating the performance of prominent models like GPT-3.5 and GPT-4, our findings underscore the potential of LLMs to enhance efficiency and accuracy in pharmacovigilance text analytics. With properly designed prompts, achieving high sensitivity is a positive indicator for literature review tasks, ensuring the identification of targeted contexts crucial to our focus. Simultaneously, the high reproducibility observed, based on different examples provided to the models, indicates the stable behavior of LLMs for reviewing and categorizing literature. While we achieve only a moderate specificity of around 70%, this is acceptable considering the low positive rate typically encountered in literature review tasks. LLMs can still effectively filter out a significant number of negative articles, saving time and efforts for reviewers.

Various elements may impact the performance of LLM-based literature screening. A properly designed pipeline, including prompts, chain-of-thought contexts, and N-shot learning scenarios can significantly benefit the accurate categorization and further assist in human review and decision-making processes. As LLMs continue to undergo rapid development, a deep understanding of the data we are working on, and the evolving landscape of AI techniques, will play a crucial role in further refining and optimizing this task.

This study contributes valuable insights to the evolving landscape of AI applications in pharmacovigilance, emphasizing the need for nuanced model evaluation, careful prompt design, and consideration of example set balance. As the intersection of AI and healthcare progresses, these findings pave the way for informed advancements in drug safety monitoring and signal detection, ultimately contributing to enhanced patient safety and the continual improvement of pharmacovigilance practices.

## 5 Discussion

### 5.1 Consistent incorrect predictions and model bias

The model prediction based on Claude2 reasoning results in good sensitivity, indicating that the model could reliably help filter out most of the irrelevant abstract. Yet, the consistent misprediction of certain abstracts, even in the face of varied testing scenarios, poses intriguing questions about potential model bias, the accuracy of original labels, or the inherent difficulty in categorizing these specific abstracts. Based on the four examples which were constantly wrongly predicted as relevant, the inherent complexity of certain abstracts appears to be the main reason contributing to this consistent discrepancy. The first discussed that the formation of a carcinogenic byproduct after chlorine disinfection, when below critical values set by the regulations, is no longer a risk to human health. The second examined the effectiveness of small doses of chlorine as a treatment of dysentery. The third suggested that the concentrations of extractable organic chlorine in sediment may be used an indicator for environmental contamination. This prompts a critical reflection on the limitations and challenges in achieving absolute precision in complex tasks. The last one discussed the chlorination byproducts of a chemical and the mutagenicity evaluation of the byproducts. These examples provided a critical reflection on the limitations and challenges in achieving absolute precision of the model in the complex tasks.

### 5.2 Effectiveness of human expert vs. model-generated reasoning

The unexpected finding that model-generated reasoning outperforms human expert-generated explanations in terms of prediction results raises intriguing questions. One possible explanation could be that models have the adaptability to underlying structures and patterns in example data, while human experts introduce nuances beyond the model’s scope. Also, human experts, despite domain expertise, might approach classification with biases or preconceptions, diverging from the model’s inherent understanding. The interpretability could be another challenge for models. The interpretability and alignment of human-generated reasoning with the model’s decision-making processes pose challenges due to subjective nature, introducing variability. This is still an open question, and future studies should refine human-AI collaboration to leverage both strengths, enhancing interpretability and effectiveness in pharmacovigilance literature screening.

### 5.3 Insights from GPT-4’s reasoning judgments

The valuable insights gleaned from GPT-4’s consistent preference for Claude2-generated reasoning provide a foundation for future directions. Besides providing an accurate predictive response, leveraging AI models to generate high-quality reasoning is an exciting avenue. The potential to distill the essence of “good reasoning” through the lens of AI models opens new possibilities for refining prompt structures and advancing the field, as the AI could learn the intelligence underlying the prompt which was provided by human (Sejnowski, 2023) or another AI. By applying the Chain-of-Thought (CoT) strategy, we can enforce the model to provide explanation on specific aspects, which can dramatically improve the reasoning abilities of LLMs. More importantly, this prompt engineering process can be both manually and automatically to maximize its capability to capture important reasoning aspects, as well as the specific needs from the reviewers.

The judgments of the reasoning by GPT-4 would be helpful for us to understand what good reasoning is, to further contribute to a deeper understanding of the criteria that define effective reasoning in the context of pharmacovigilance. Next, we plan to use AI models to summarize and optimize the prompt to generate good reasoning and how to identify if an abstract is relevant or not more correctly.

### 5.4 Identified factors influencing prediction results

Through extensive testing, we identified several influential factors shaping prediction outcomes. Notably, the choice of prompt, a recognized driver of AI responses, proved crucial in our study. The models are sensitive to specific keywords, such as “directly”. In our prompt4, most of the relevant abstracts were predicted as irrelevant by GPT-3.5 when “directly related” was added. The finding highlighted the nuanced interplay between prompts and model responses. GPT-4 appeared to be more sensitive to terms with specific definitions, such as “safety” and “toxicity,” which in turn influenced the model’s sensitivity and specificity. Notably, “safety” is a broader concept than “toxicity,” which may explain some of the variation in the model’s performance.

Other than the five prompts we showcased in the main results, we tested various prompts that led to worse, sometimes significantly biased results. For instance, with the prompt “Read the abstract, decide whether it reports the safety of chlorine or chlorine base compounds.” for GPT-3.5, we obtained a median specificity of 0.95. However, the sensitivity was dropped to as low as 0.2, resulting in an F1 score of 0.31. Clearly, the model tended to predict every sample as negative.

Another unexpected discovery that emerged during our analysis was some formatting in the prompt. Introducing specific language cues, such as “please provide me the label as [relevant] or [irrelevant],” unintentionally influenced the prediction outcomes. This alteration significantly increased the number of categories predicted as [relevant], demonstrating the model’s sensitivity but resulting in low specificity.

Additionally, we identified the balance within example sets (the example abstracts, labels, and reasoning we provided to the models) as a critical factor shaping prediction outcomes. The introduction of unbalanced sets—comprising either all positives or all negatives—led to a notable shift in sensitivity and specificity. In our study, this imbalanced example set consistently resulted in increased specificity and decreased sensitivity, indicating that the models encountered difficulty in correctly identifying relevant abstracts.

Although we observed promising results of LLMs for literature screening in this study, these results are limited by the specific dataset used, which may not represent other domains. It is valuable to extend this study across multiple datasets from diverse domains to more robustly demonstrate their generability in the future. Meanwhile, the effectiveness of LLMs heavily depends on the quality of the prompts provided. Poorly constructed prompts can lead to bad performance, highlighting the need for end users to develop skills in precise and effective prompting. Therefore, LLMs should be used as tools to augment human capabilities, with a clear awareness of their limitations and proper prompting techniques. These factors introduce complexity to the prediction process, highlighting the importance of meticulous prompt design and example set structuring for optimal and reliable model performance. Further exploration of these factors is vital for enhancing the robustness of AI systems across diverse contexts.

A notable limitation of using LLMs for health-related literature reviews, or any specific domains, is their training on domain-neutral texts rather than specialized medical literature, which can impact the accuracy and comprehensiveness of the reviews. Models may not always perform well without further optimization. To mitigate this, one effective approach is providing detailed contexts in the prompts as background information or examples, which has shown to improve the model’s understanding and processing of specialized content. Additionally, fine-tuning LLMs on medical-specific datasets, although not tested in this study due to high costs, holds potential for significantly enhancing their performance in health-related use cases. These strategies highlight the importance of adapting LLMs to better meet the demands of specialized domains like healthcare.

### 5.5 High sensitivity is critical to the task

The heightened sensitivity (95%) provides a strategic advantage, enabling the models to accurately identify literature relevant to our interests. This ensures that important contextual information is not overlooked. On the other hand, we can somehow tolerate moderate (70%) specificity since the positive rates in most of the literature screening tasks are low, which means most of the articles collected are irrelevant and need to be filtered out. In a scenario where 10,000 abstracts are collected in a project with a positive rate of 15%, 8,500 irrelevant abstracts would need to be reviewed by reviewers, consuming considerable time and effort. With the assist of LLMs, 5,950 irrelevant abstracts (70% of 8,500) could be filtered out. This streamlined workload allows human experts to focus on a more refined selection of articles, enhancing the overall efficiency of drug safety monitoring.

### 5.6 Consideration of open-source models

In our study, we focused on evaluating the performance of specific LLMs, including GPT-3.5, GPT-4, Claude2, based on their prominence and availability at the time of our research. We recognize that there are other efficient models, such as Llama and Mixtral, which are open-source and could be potential alternatives. While these models are open-source, deploying them on local machines poses challenges. Significant computing resources and technical expertise such as programming skills for setup and maintenance are required. As a result, these models were not included in our current study. Future research could explore the performance and cost-effectiveness of a broader range of models, including open-source options, to provide a more comprehensive understanding of the potential of LLMs in pharmacovigilance literature screening.

## Data Availability

The original contributions presented in the study are included in the article/Supplementary Material, further inquiries can be directed to the corresponding author.

## Supplementary material

SUPPLEMENTARY FIGURE S1 Evaluation of prediction results using GPT-4. Five-shot learning and reasoning explanations generated by Claude2 were applied.
