# Supplementary figures and images for: Assessing the performance of large language models in literature screening for pharmacovigilance: a comparative study
Source: Front Drug Saf Regul. 2024 Jun 27;4:1379260. doi: 10.3389/fdsfr.2024.1379260 (PMC12443093; doi:10.3389/fdsfr.2024.1379260)

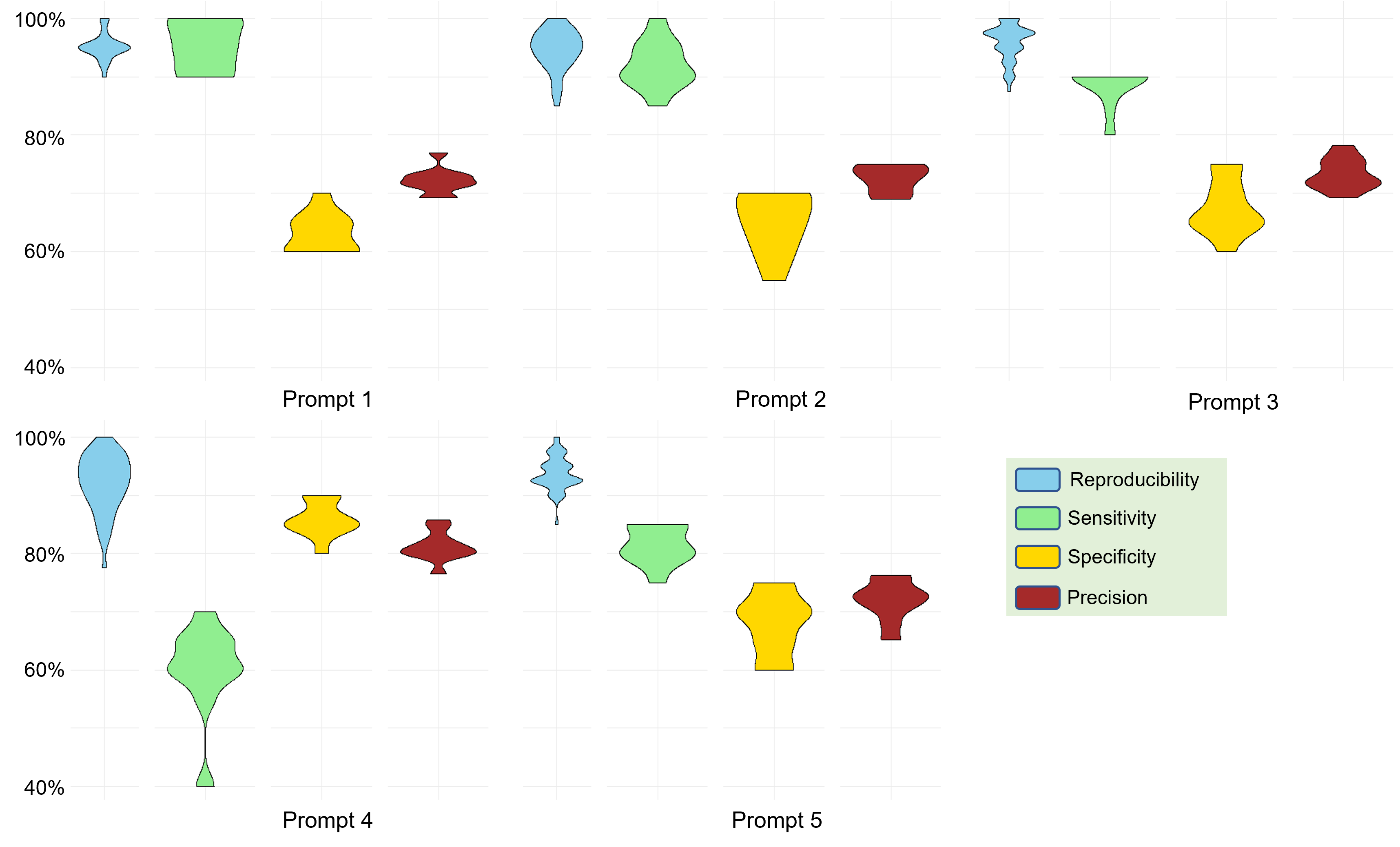

Supplement: Supplementary file 1 [file Image1.tif]
