# Supplementary material for: Assessing the performance of large language models in literature screening for pharmacovigilance: a comparative study
Source: Front Drug Saf Regul. 2024 Jun 27;4:1379260. doi: 10.3389/fdsfr.2024.1379260 (PMC12443093; doi:10.3389/fdsfr.2024.1379260)
Supplement: Supplementary file 2 [file DataSheet1.docx]

Supplementary Material

# Supplementary Data

The full prompt used in this study.

{'role': 'system', 'content': 'You are an AI model trained to understand and summarize abstracts.'},

{'role': 'user', 'content': 'I am conducting a study on chlorine safety within a regulatory scenario (or any compound of/related to/containing chlorine), aiming to assess its antiseptic efficacy, toxicity, and potential safety issues. Specifically, I seek to identify relevant abstracts that provide insights into human toxicity, side effects, and related aspects, as well as to distinguish irrelevant abstracts that are not pertinent to the study of chlorine safety.'},

{'role': 'system', 'content': 'Please study the following abstracts, their categories of relevant or irrelevant, and their corresponding reasoning explanations.'}

{'role': 'assistant', 'content': f'Category: [{categories[a]}]; Abstract: [{abstracts[a]}]; Reasoning explanation: [{reasonings[a]}]'} #the examples were provided to the model in a loop, the number of loops depended on the numbers of shots.

{'role': 'user', 'content': f'{question}'} #the question was one of the five listed in Figure 4.

This first part of the prompt remained the same and was provided to the models together with the questions listed in Figure 4. We added this explanation as a supplementary file.
